# *Punica granatum* L. Extract Shows Cardioprotective Effects Measured by Oxidative Stress Markers and Biomarkers of Heart Failure in an Animal Model of Metabolic Syndrome

**DOI:** 10.3390/antiox12061152

**Published:** 2023-05-25

**Authors:** Joanna Niewiadomska, Monika Kasztura, Izabela Janus, Elżbieta Chełmecka, Dominika Marta Stygar, Piotr Frydrychowski, Aneta Wojdyło, Agnieszka Noszczyk-Nowak

**Affiliations:** 1 Department of Internal and Diseases with Clinic for Horses, Dogs, and Cats, Faculty of Veterinary Medicine, Wroclaw University of Environmental and Life Sciences, 50-375 Wrocław, Poland; piotr.frydrychowski@upwr.edu.pl; 2 Department of Food Hygiene and Consumer Health Protection, Wroclaw University of Environmental and Life Sciences, 50-375 Wrocław, Poland; monika.kasztura@upwr.edu.pl; 3 Department of Pathology, Division of Pathomorphology and Veterinary Forensics, Faculty of Veterinary Medicine, Wroclaw University of Environmental and Life Sciences, C. K. Norwida 31, 50-375 Wrocław, Poland; izabela.janus@upwr.edu.pl; 4 Department of Statistics, Department of Instrumental Analysis, Faculty of Pharmaceutical Sciences in Sosnowiec Medical University of Silesia, 40-751 Katowice, Poland; echelmecka@sum.edu.pl; 5 Department of Physiology, Faculty of Medical Sciences in Zabrze, Medical University of Silesia, 40-751 Katowice, Poland; dstygar@sum.edu.pl; 6 Department of Fruit, Vegetable and Nutraceutical Plant Technology, Wrocław University of Environmental and Life Sciences, 51-630 Wrocław, Poland; aneta.wojdylo@upwr.edu.pl

**Keywords:** *Punica granatum* L. peels, cardioprotection, oxidative stress, metabolic syndrome

## Abstract

Metabolic syndrome (MetS) significantly increases the risk of cardiovascular diseases (CVD), a leading cause of death globally. The presented study investigated the cardioprotective role of dietary polyphenols found in pomegranate peels in an animal model of metabolic syndrome. Zucker diabetic fatty rats (ZDF, MetS rats, fa/fa) were supplemented with polyphenol-rich pomegranate peel extract (EPP) at two dosages: 100 mg/kg BW and 200 mg/kg BW. The extract was administered for 8 weeks. The effect of ethanolic peel extract on the concentration of oxidative stress markers (CAT, SOD, MnSOD, GR, GST, GPx, TOS, SH, and MDA), biomarkers of heart failure (cTnI, GAL-3), and alternations in tissue architecture was assessed. The results showed a significant increase in SH concentration mediated via EPP supplementation (*p* < 0.001). Treatment with a 100 mg/kg BW dosage reduced the TOS level more efficiently than the higher dose. Interestingly, the CAT and GST activities were relevantly higher in the MetS 100 group (*p* < 0.001) compared to the MetS control. The rats administered EPP at a dose of 200 mg/kg BW did not follow a similar trend. No differences in the GR (*p* = 0.063), SOD (*p* = 0.455), MnSOD (*p* = 0.155), and MDA (*p* = 0.790) concentration were observed after exposure to the pomegranate peel extract. The administration of EPP did not influence the cTnI and GAL-3 levels. Histology analysis of the heart and aorta sections revealed no toxic changes in phenolic-treated rats. The findings of this study prove that the extract from pomegranate peels possesses free radical scavenging properties in the myocardium. The effect on alleviating ventricular remodeling and cardiomyocyte necrosis was not confirmed and requires further investigation.

## 1. Introduction

Cardiovascular diseases (CVDs) are still the leading cause of mortality worldwide [1,2]. This group of heart and blood vessel disorders comprises principally coronary heart disease, peripheral arterial disease, cerebrovascular disease, and thrombo-embolic disease [3]. A crucial part of preventing CVDs is lifestyle modification, especially based on developing healthy eating patterns and physical activity. To control the burden of CVD-related mortality and disability, clinical strategies aimed at declining modifiable risk factors, including dyslipidemia, hypertension, diabetes, alcohol consumption, smoking, and obesity, must be implemented [4]. However, the global trend of increasing cardiovascular risk factors in most world regions has been observed, mainly linked to an epidemic of obesity [5].

One cluster of pathologies that significantly contributes to increased heart disease, stroke, and diabetes is metabolic syndrome (MetS). These conditions include central obesity, elevated blood pressure, hyperglycemia, insulin resistance, and dyslipidemia [6,7]. Each constitutes a single risk factor conducive to cardiovascular disorders. However, their co-occurrence increases the risk to an extremely high level. Obesity is believed to play a prominent role in MetS pathogenesis, and constitutes a trigger agent for other components. MetS is also associated with pro-thrombotic and proinflammatory states [8]. Cardiovascular changes occurring in MetS comprise a multifaceted image of overlapping pathologies. Obesity-associated alternations in heart muscle anatomy and function have been defined as “cardiomyopathy of obesity” [9]. The cardiac phenotype in obesity comprises concentric left ventricular hypertrophy, diastolic and systolic dysfunction, myocardial fibrosis, and microvascular dysfunction. Finally, the deterioration in myocardial function leads to heart failure [10,11]. Using biomarkers in the detection of heart impairment seems to be pivotal in verifying cardiac acute and chronic alternations. One such biomarker that is associated with the disruption of myocardial integrity is troponin I (cTnI), reflecting acute cardiac injury [12,13]. Galectin-3 (GAL-3), in turn, can be used to obtain insight into the progression and severity of myocardial architecture remodeling, including fibrosis [14,15].

Polyphenols are secondary metabolites produced by plants through metabolic pathways acting as protective agents against biotic and abiotic stresses and contributing to plants’ growth and development [16,17]. The nutrient value of phytochemicals is guaranteed by antioxidant, anti-inflammatory, antibacterial, antifungal, antiallergic, antiangiogenic, anticoagulant, immunomodulatory, anticancerogenic, and antimutagenic properties [18,19]. Previously conducted studies have proven that the supplementation of phenolic compounds can mitigate or even reverse the pathological changes in cardiovascular diseases (coronary heart disease, stroke, heart failure, and thrombo-embolic disease) and endocrine disorders (diabetes mellitus and osteoporosis) [20,21].

The parley on the cardiovascular protective role of polyphenols remains open. The relevancy of polyphenol intake and CVD outcomes has been investigated in many studies. Some natural sources of phenolic compounds and individual particles, such as resveratrol, purple grapes, green tea, and coca, exert a favorable effect on cardiovascular health [21]. Phenolic compounds’ mechanism of action in reference to cardiovascular protection comprises the inhibition of platelet aggregation and activation; boosting of the bioavailability of nitric oxide for the endothelium, resulting in vasodilatation; free radical scavenging properties; and the inhibition of LDL oxidation [18,22].

Pomegranate (*Punica granatum* L.) has been used as a traditional remedy for centuries in the Middle East. Different parts of the plant contain various phytochemicals. In particular, fruit consumed as juice, pulp or beverage was believed to possess more bioactive compounds than the non-editable parts. However, health benefits are also attributed to peels, leaves, or seeds [23,24]. It has been proven that pomegranate peels, a by-product of juice extraction, are abundant in phenolic compounds such as flavonoids, ellagitannins, and anthocyanidins, and metabolites such as urolithins [25]. Compared to the juice and seeds, pomegranate peel extract exhibits comparable antioxidant and anti-inflammatory activity. Moreover, it also displays antidiabetic, antiatherosclerotic, antihypertensive, antihyperlipidemic, antimicrobial, and anticancer properties [26]. In this context, peels appear to be valuable biowaste products with promising biological and pharmaceutical activities.

The present study focused on the role of dietary polyphenols found in pomegranate peels as cardiovascular protective agents regarding the oxidative status in heart tissue, the level of biomarkers of heart failure, and histopathologic changes occurring in the heart and aorta in an animal model of metabolic syndrome.

## 2. Materials and Methods

### 2.1. Animals and Diet

The study project was approved by the Ethics Committee for Experiments on Animals at the Ludwik Hirszfeld Institute of Immunology and Experimental Therapy Polish Academy of Sciences, Wroclaw, Poland (Resolution 53/2017). Zucker diabetic fatty rats (metabolic syndrome rats (MetS), ZDF-Leprfa/Crl, fa/fa) were purchased from Sulzfeld (Charles River Laboratories, Research Models and Services, Germany GmbH). The rats were housed in groups of two per cage and maintained on a 12 h light–dark cycle at a temperature of 20 °C ± 2 °C and a relative humidity of 40–60%. All animals had free access to water and Purina 5008 feed (LabDiet, Charles River Laboratories, Wilmington, MA, USA).

### 2.2. Extraction Procedure of Pomegranate Peel Phenolic Compounds

The fresh pomegranate fruit *Punica granatum* L. (cultivar Mollar de Eche) was delivered from Spain. The peels were collected, dried, and shredded in Thermomix. Subsequently, the obtained peel powder in the total amount of 1 kg was extracted and reextracted twice with 50% ethanol in an ultrasonic bath for 25 min. After the extraction procedure, the mixtures were centrifugated, and the supernatants were filtered. The resulting filtrate was concentrated by a Rotavapor rotary evaporator in a water bath at 40 °C. The concentrated extract from pomegranate peel was passed through the column filled with Amberlite XAD-16 resin (Brenntag, Essen, Germany) and eluted with distilled water to remove the organic acids, sugars, and other compounds. The polyphenol elution procedure was carried out with an 80% aqueous ethanol solution. Afterward, the gathered fractions were dried in an SPT-200 vacuum oven (Zeamil, Krakow, Poland).

### 2.3. Identification of Polyphenolic Compounds by LC-PDA—QTOF/MS and Quantification via the UPLC-PDA Method

The identification and quantification of polyphenols from pomegranate peels were performed with the use of an Acquity ultra-performance liquid chromatography (UPLC) system, consisting of a photodiode array detector (PDA; Waters Corporation, Milford, MA) coupled with a G2 mass detector quadrupole time-of-flight (Q-TOF) MS instrument (UPLC/Synapt Q-TOF MS, Waters Corp., Milford, CT, USA), equipped with an electrospray ionization (ESI) source. Separation was carried out on the Acquity BEH C18 column (100 mm × 2.1 mm, 1.7 µm, Waters Corp., Milford, CT, USA). The samples were dissolved in MeOH/H_2_O/ascorbic acid (30:68:1, *v*/*v*/*m*) with a 1% hydrochloric acid (37%) mixture. The volume injected was 10 µL. The mobile phase was composed of a mixture of 0.1% (*v*/*v*) aq. formic acid (A) and acetonitrile (B). The gradient program was set as follows: the initial phase consisted of 99% of A for 1 min; subsequently, an 11 min linear gradient of 1% to 40% of B was applied, and after 12 min, B was increased to 100%, continued to 2 min, and then returned to inductive conditions (99% of A) at a 2 min step. For elution, a mobile phase flow rate of 0.45 mL/min was used. The operating parameters for the Q-TOF MS were as follows: capillary voltage of 2.5 kV, cone voltage of 30 V, cone gas flow of 11 L/h, collision energy of 28–30 eV, source temperature of 100 °C, desolvation temperature of 250 °C, collision gas, argon, desolvation gas (nitrogen) flow rate of 300 L/h, data acquisition range of m/z 100–2000 Da, and a negative ionization mode. The data from LC-MS were analyzed with the MassLynx 4.0 ChromaLynx Application Manager software. Individual phenolic compounds were characterized via the retention time and exact molecular masses. Identification was based on MS/MS analysis and comparison of the obtained results with literature data. The quantification of the derivatives of ellagic acid was performed using UPLC-PDA. The spectra were measured in the 200–600 nm wavelength range in steps of 2 nm. The findings are presented as milligrams per 100 g dry matter (dm).

### 2.4. Experimental Design

**Figure 1:**
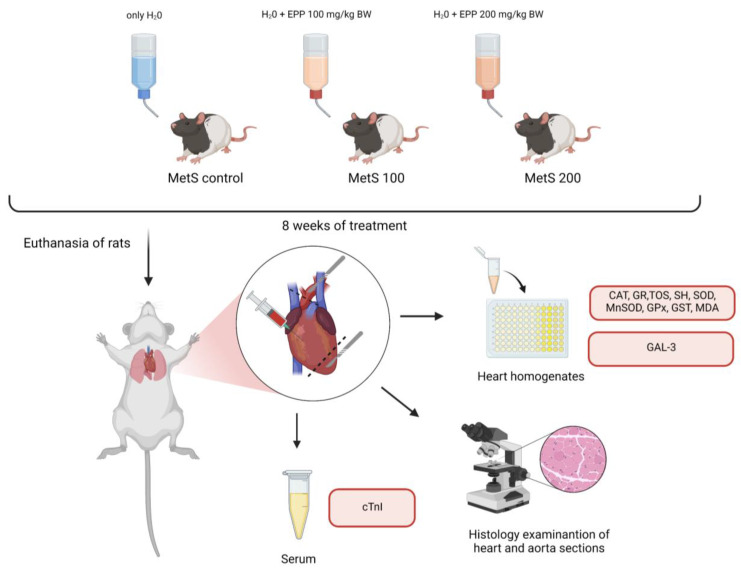
Experimental design. This scheme was created using Biorender.com (accessed on 11 May 2023). Abbreviations: EPP—extract from pomegranate peels, BW—body weight, MetS—rats with a mutation in the leptin receptor gene predisposed to metabolic syndrome, CAT—catalase, GR—glutathione reductase, TOS—total oxidative status, SH—protein thiol groups, SOD—superoxide dismutase, MnSOD—Mn-dependent superoxide dismutase, GPx—glutathione peroxidase, GST—glutathione transferase, MDA—malondialdehyde, GAL-3—galectin 3, and cTnI—troponin I.

After 2 weeks of acclimatization, the rats were divided into three groups as follows: (1) MetS control—these control-group (ZDF) rats received only water; (2) MetS 100—this study group (ZDF) received an extract from pomegranate peels (EPP) at a dose of 100 mg/kg BW; and (3) MetS 200—this study group (ZDF) received EPP at a dose of 200 mg/kg BW. Each group consisted of six animals. The extract from the pomegranate peels was administered daily by oral gavage using water as a vehicle. During the study, body weight in all groups increased pointedly, reaching 407.14 ± 32.68 g in MetS control, 382.08 ± 35.48 g in MetS 100, and 392.75 ± 36.28 g in Mets 200 by the end of the study. The performed biochemistry panel assessing the glycemic and lipid profile proved the hyperglycemic and hyperlipidemic status of the animals used in the study. After 8 weeks, the rats were fasted overnight and euthanized via an intraperitoneal injection of pentobarbital. Before the termination procedure, every individual was sedated using a mixture of intramuscular anesthetics as follows: ketamine at a dose of 60 mg/kg and medetomidine at a dose of 0.3 mg/kg. The heart and aorta samples were subsequently harvested. The dissected tissues were immersed in liquid N_2_ and subsequently stored at −80 °C. Serum samples were collected at three consecutive time points: before EPP administration, after 4 weeks, and after 8 weeks of study (Figure 1).

### 2.5. Preparation of Homogenates from Heart Tissue

The fragments of cardiac tissue from the left ventricle were weighted (about 100 mg) and homogenized (1:10 *w*/*v*) in PBS with protease inhibitors using tissueruptor (Qiagen). The lysates were then incubated for 10 min on ice cold water and centrifuged for 15 min at 14,000× *g* at 4 °C. The protein concentration in the supernatants was assessed according to the Lowry method [27]. The protein samples were frozen and stored in liquid nitrogen for further analysis.

### 2.6. Assessment of Oxidative Stress Markers

The evaluation of antioxidant capacity was analyzed in the heart tissue. The following biomarkers were determined: the activity of total catalase (CAT, IU/g protein), superoxide dismutase (SOD, NU/mg protein), glutathione reductase (GR, IU/g protein), glutathione transferase (GST, IU/g protein), glutathione peroxidase (GPx, IU/g protein), and Mn-dependent superoxide dismutase (MnSOD, NU/mg protein). Moreover, the protein thiol groups (SH, µmol/g protein), to indicate the redox potential and total oxidative status (TOS, µmol/g protein), were estimated to assess the entire antioxidants in the samples. The malondialdehyde (MDA) concentration was used as a marker of lipid peroxidation secondary to increased oxidative stress. For the measurement of the change in absorbance in spectral assays, a Perkin Elmer Victor X3 reader was applied (PerkinElmer, Inc., Waltham, MA, USA).

#### 2.6.1. Catalase Activity (CAT, EC 1.11.1.6)

Catalase activity was assessed following the Aebi protocol [28]. Following the method principles, the heart tissue homogenate was mixed with phosphate buffer (50 mM TRIS/HCl), and subsequently, the reaction was initiated by adding hydrogen peroxide (H_2_O_2_). The enzyme activity was measured using the UV spectrophotometric method, monitoring the alternation of 240 nm absorbance, as the decomposition of H_2_O_2_ is followed by a decrease in absorbance. CATactivity was displayed per 1 g of protein (IU/g protein).

#### 2.6.2. Superoxide Dismutase Activity (SOD, EC 1.15.1.1)

Superoxide dismutase activity was determined using the Oyanagui method [29]. In this protocol, xanthine oxidase catalyzes the reaction, generating the superoxide anion free radical (O_2_^−^), which oxidases hydroxylamine (which turns into the nitrile form). The nitro anion, together with a chromogenic agent, gives a colored product. In the presence of SOD in samples, the reaction of the formation of nitro anion is reduced because of the inhibitory effect of the enzyme on the superoxide anion-free radical. The SOD activity was measured spectrophotometrically at 550 nm and expressed as nitrile units (NU) per 1 mg of protein (NU/mg protein).

MnSOD activity was assessed by adding potassium cyanide to the reaction mixture, which is responsible for the inactivity of CuZnSOD. The total MnSOD activity was presented in nitrile units per 1 mg of protein (NU/mg protein).

#### 2.6.3. Glutathione Reductase Activity (GR, EC 1.8.1.7)

GR activity was determined using the kinetic method described by Calberg and Manervick [30]. The core of the method is based on the NDPH-dependent reduction of glutathione disulfide (GSSG) to glutathione (GSH). The produced beta-nicotinamide dinucleotide phosphate (NDPH) as a hydrogen donor in the regeneration of GSH was measured spectrophotometrically at 340 nm. GR activity was presented per 1 g of protein (IU/g protein).

#### 2.6.4. Glutathione Peroxidase Activity (GPx, EC 1.11.1.9)

GPx activity was assessed using the kinetic method, according to Mannervik [31]. The enzyme catalyzes the reduction of hydroperoxide (ROOH) coupling with the oxidation of glutathione (GSH) to glutathione disulfide (GSSG), and beta-nicotinamide dinucleotide phosphate (NDPH) to nucleoside-diphosphate (NDP). In this method, the decrease in NDPH absorbance measured at 340 nm correlated with GPx activity, which was expressed per 1 g of protein (IU/g protein). As a substrate, tert-Butyl hydroperoxide was used.

#### 2.6.5. Glutathione S-Transferase Activity (GST, EC 2.5.1.18)

GST activity was measured using the kinetic method established by Habig and Jakoby [32]. In this spectral assay of the reduced glutathione and analyzed sample, the substrate 1-chloro-2,4-dinitrobenzene was added. The principle of the method arises from the change in a specific quantity of substrate after its conjugation with glutathione (GSH). After incubating the reaction mixture at room temperature, the change in absorbance at 340 nm was measured. GST activity was displayed per 1 g of protein (IU/g protein).

#### 2.6.6. Protein Thiol Groups (SHs)

Protein thiol group (SH) concentration was determined according to Sedlak and Lindsay [33]. This spectrophotometric protocol is based on Elman’s reagent, 5,5′-dithiobis-(2,-nitrobenzoic acid), i.e., DTNB. Aliquot tissue homogenates were mixed with tris buffer and DTNB. Subsequently, the proteins were precipitated by incubation with methanol. The absorbance of relatively clear supernatants was read at 412 nm, and the SH concentration was presented in µmol/g protein.

#### 2.6.7. Total Oxidative Status (TOS)

Total oxidative status (TOS) determination followed the colorimetric method according to Erel [34]. The technique is grounded on the oxidation of the ferrous ion–o-dianisidine complex to ferric ion by the oxidants in the analyzed sample. In an acid medium, the produced ferric ion reacts with xylenol orange, making a colored complex that can be measured spectrophotometrically at a 560 nm wavelength. Hydrogen peroxide was used for calibration. The TOS result was expressed in µmol/g protein.

#### 2.6.8. Malondialdehyde Concentration (MDA)

Malondialdehyde concentration was determined using the previously described protocol by Ohkawa et al. [35]. The procedure of assessing lipid peroxides in animal tissue is based on their reaction with thiobarbituric acid. As the pH affects the reagents mixture, the pH value was set at 3.5. The measurements were conducted spectrophotometrically and compared with a standard curve. Tetramethoxypropane (TMP) was used as the external standard. The MDA concentration was displayed in µmol/g protein.

### 2.7. Assessment of Heart Failure Biomarkers

Galectin-3 (GAL3, pg/mL) and troponin I (cTnI, pg/mL) were used as the biomarkers of heart failure. These proteins correlate with heart muscle remodeling and impairment, including fibrogenesis, inflammation, and myocardial necrosis. Commercial ELISA assays were applied.

#### 2.7.1. Galectin-3 (GAL-3)

Galectin-3 concentration in heart tissue homogenates was measured using a quantitative Rat Galectin-3 (LGALS3) ELISA Kit (Thermo Fisher Scientific, Waltham, MA, USA). The myocardial tissue samples were homogenized in PBS and centrifugated. An ELISA assay was carried out according to the manufacturer’s instructions. GAL-3 in the analyzed samples, after incubating with immobilized monoclonal antibodies, was detected using biotin-conjugated anti-GAL3 polyclonal antibodies. Then, streptavidin horseradish peroxidase and 3,3′,5,5′-tertamethylbenzidine (TMB) were added to initiate the reaction. The absorbance was read within 30 min after adding the stop solution at 450 nm, and the GAL-3 concentration was calculated based on the standard curve and expressed in pg/mL.

#### 2.7.2. Troponin T (cTnI)

Troponin I in the serum was assessed using quantitative the Rat Cardiac Troponin I (ab246529) ELISA Kit (Abcam, Eugene, OR, USA), applying the manufacturer’s protocol. After adding to appropriate wells, the samples and standards were incubated with an antibody cocktail for an hour. The reaction was developed using a 3,3′,5,5′-tertamethylbenzidine (TMB) substrate solution. The results were read on a microplate reader set at a wavelength of 450 nm. The cTnI concentration was determined by interpolating the absorbance values against the standard curve and presented in pg/mL.

### 2.8. Histological Analysis

Histology examination was performed on the heart and aorta tissue samples. The specimens were fixed in 7% buffered formalin, dehydrated, embedded in paraffin blocks, and cut into 6 µm sections. The sections were stained with hematoxylin and eosin (HE), Masson-Goldner trichrome (MGt), and Picro Sirius Red stain (PSR). The heart and aorta specimens were evaluated on a semi-quantitative scale from 0 (no changes) to 3 (severe changes). The examined changes in the heart samples included the presence and intensity of parenchymal degeneration, the presence and intensity of fibrosis, and the presence and intensity of inflammatory infiltration. In the specimens obtained from the aorta, the fiber disarrangement was evaluated.

### 2.9. Statistical Analysis

The data are displayed as the mean ± standard deviation (SD) for biochemical analysis or median (min–max) for histological analysis. Statistical analyses were conducted using Statistica 12.5 PL. The one-way parametric ANOVA, followed by post hoc Dunnett testing was applied for variables with a normal distribution. The figures show the mean values and 95% confidence intervals. The distribution and variance homogeneity were evaluated using the Shapiro–Wilk and Levene’s tests, respectively. The outliers in the data were detected and not considered in statistical analysis; *p* < 0.05 was considered significant.

## 3. Results

### 3.1. Identification and Quantification of Phenolic Compounds in Pomegranate Peel Extract

Ellagic acid and its derivatives were found in the analyzed sample of extract from pomegranate peels. Among the 14 ellagic acid derivatives, the most abundant were: punicalagin isomer (R_t_ = 2.12 min), HHDP-gallagyl-hexoside (R_t_ = 2.87 min), HHDP-gallagyl-hexoside (R_t_ = 3.69 min), ellagitannin (R_t_ = 5.08 min), and ellagic acid-hexoside (R_t_ = 6.08 min). Hydrolyzable tannins possessed a higher concentration and seemed to be the main antioxidant compound in pomegranate peels. The results of the analysis are presented in Table 1.

### 3.2. Impact on Oxidative Stress Markers

**Figure 2:**
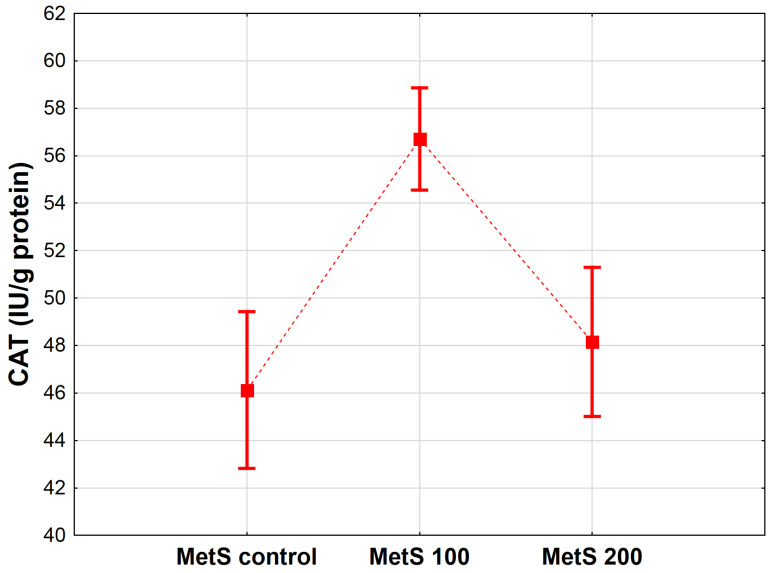
Mean catalase activity (CAT, IU/g protein) in heart homogenates of rats from the groups administered only water, as well as the extract from pomegranate peels (EPP) at a dose of 100 mg/kg BW, or 200 mg/kg BW. Groups: MetS control (only water), MetS 100 (EPP at a dose of 100 mg/kg BW), and MetS 200 (EPP at a dose of 200 mg/kg BW). Vertical lines represent 95% confidence intervals, and the points reflect the mean of the group. For the reader’s convenience, the markers were connected by dashed lines.

There were significant differences in myocardial CATactivity between the groups (Figure 2). Statistical analysis exhibited that in the groups with MetS, the CAT concentration was higher in the experimental group MetS 100 treated with 100 mg/kg EPP than in the control MetS group (*p* < 0.001). No statistical significance was observed in the experimental group MetS 200 treated with 200 mg/kg EPP in comparison to rats receiving only water (*p* < 0.364).

**Figure 3:**
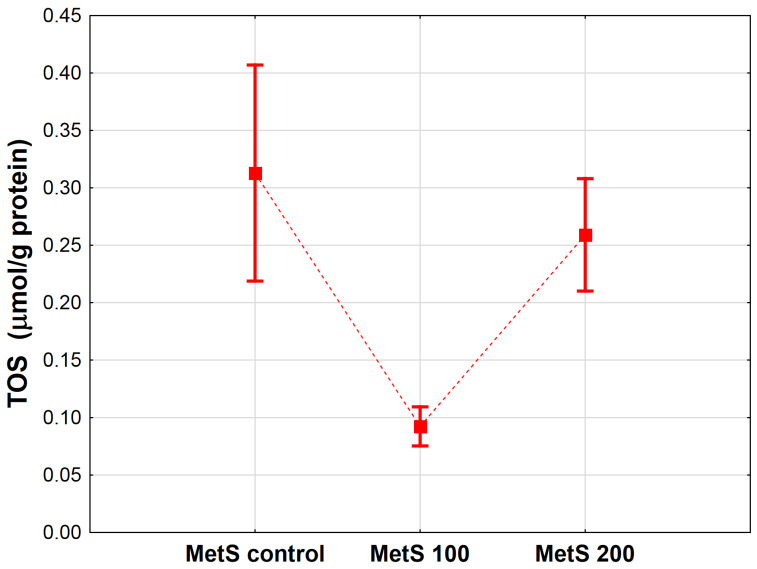
Mean total oxidative status (TOS, µmol/g protein) in heart homogenates of rats from the groups administered only water, as well as the extract from pomegranate peels (EPP) at a dose of 100 mg/kg BW, or 200 mg/kg BW. Groups: MetS control (only water), MetS 100 (EPP at a dose of 100 mg/kg BW), and MetS 200 (EPP at a dose of 200 mg/kg BW). Vertical lines represent 95% confidence intervals, and the points reflect the mean of the group. For the reader’s convenience, the markers were connected by dashed lines.

The TOS concentration was decreased in rats with MetS treated with 100 mg/kg EPP (MetS 100) in comparison to the control group (MetS control, *p* < 0.001). No explicit differences between the control group and the rats supplemented with 200 mg/kg were found (*p* = 0.227). The results are presented in Figure 3.

**Figure 4:**
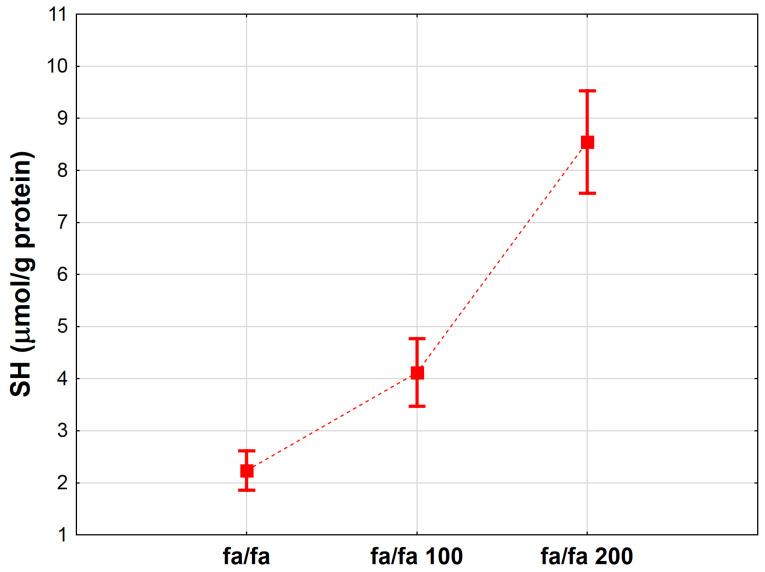
Mean protein thiol group concentration (SH, µmol/g protein) in heart homogenates of rats from the groups administered only water, as well as the extract from pomegranate peels (EPP) at a dose of 100 mg/kg BW or 200 mg/kg BW. Groups: MetS control (only water), MetS 100 (EPP at a dose of 100 mg/kg BW), and MetS 200 (EPP at a dose of 200 mg/kg BW). Vertical lines represent 95% confidence intervals, and the points reflect the mean of the group. For the reader’s convenience, the markers were connected by dashed lines.

As shown in Figure 4, a higher extract dosage led to a significant elevation in the SH concentration. A phenolic extract dosage of 200 mg/kg compared to the control group resulted in an upswing in free sulfhydryl compounds, which correlated with antioxidant capacity in MetS rats (*p* < 0.001). Correspondingly, a significant variation was noted between the MetS rats from the group treated with 100 mg/kg EPP and the control group (*p* < 0.001).

**Figure 5:**
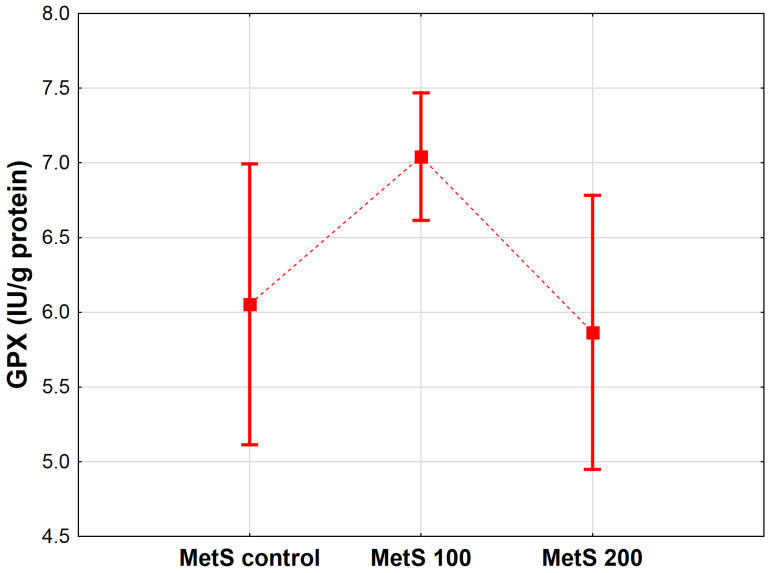
Mean glutathione peroxidase concentration (GPx, IU/g protein) in heart homogenates of rats from groups administered only water, as well as the extract from pomegranate peels (EPP) at a dose of 100 mg/kg BW or 200 mg/kg BW. Groups: MetS control (only water), MetS 100 (EPP at a dose of 100 mg/kg BW), and MetS 200 (EPP at a dose of 200 mg/kg BW). Vertical lines represent 95% confidence intervals, and the points reflect the mean of the group. For the reader’s convenience, the markers were connected by dashed lines.

EPP supplementation also affected GPx activity in the analyzed samples (*p* < 0.05). According to the results, the GPx content showed an upward tendency in the group of MetS rats treated with a lower dose (MetS 100) compared to the MetS control (*p* = 0.071). No alternations were detected in phenolic-treated MetS groups exposed to higher doses versus the MetS control (MetS 200 vs. MetS control, *p* = 0.876). A graphical representation of the obtained data is displayed in Figure 5.

**Figure 6:**
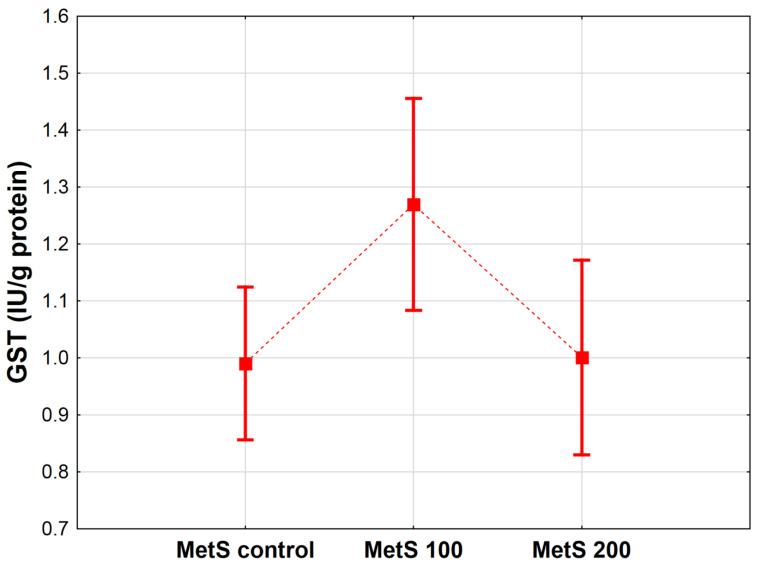
Mean glutathione transferase concentration (GST, IU/g protein) in heart homogenates of rats from groups administered only water, as well as the extract from pomegranate peels (EPP) at a dose of 100 mg/kg BW or 200 mg/kg BW. Groups: MetS control (only water), MetS 100 (EPP at a dose of 100 mg/kg BW), and MetS 200 (EPP at a dose of 200 mg/kg BW). Vertical lines represent 95% confidence intervals, and the points reflect the mean of the group. For the reader’s convenience, the markers were connected by dashed lines.

The results displayed in Figure 6 also revealed changes in GST activity in exposure to pomegranate peel extract (*p* < 0.05). Likewise, the GPx content and GST showed a significant difference only in the group of rats administered EPP at a dose of 100 mg/kg BW versus the MetS control, and the same uptrend in GST activity was observed (*p* < 0.05). In the rats treated with 200 mg/kg BW EPP, no statistically substantial variation was observed (MetS 200 vs. MetS control, *p* = 0.991).

No differences in the GR (*p* = 0.063), SOD (*p* = 0.455), MnSOD (*p* = 0.155), and MDA (*p* = 0.790) concentration were observed after exposure to pomegranate peel extract. The differences in oxidative stress markers by the group are presented in Table 2.

### 3.3. Changes in Serum Troponin I Levels (cTnI) and Galectin-3 (GAL-3) under EPP Administration

The troponin I level did not change in phenolic-treated rats (Table 3). The cTnI level in all groups was markedly elevated, which corresponds to heart injury in the course of obesity. The concentration of galectin-3, a marker of fibrosis and an indicator of myocardial remodeling, also did not change relevantly in exposure to polyphenolic extract between the groups.

### 3.4. Effect of EPP on the Heart and Aorta Tissue Composition

**Figure 7:**
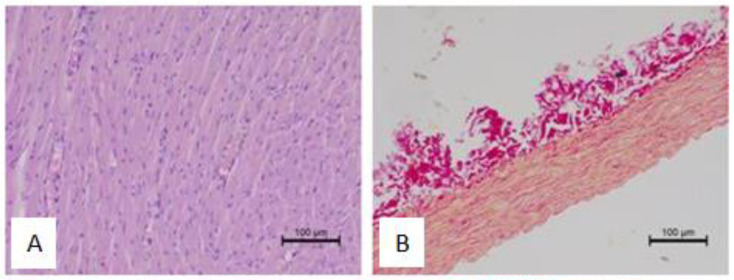

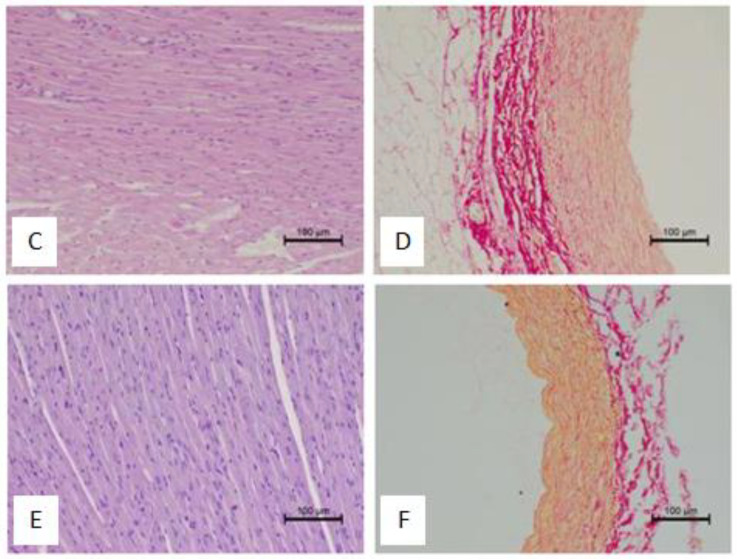
Hematoxylin–eosin-stained sections of the heart and picrosirius red staining of the aortic sections sampled from rats in various experimental groups: (**A**) heart, MetS control—MetS rat receiving only water; (**B**) aorta, MetS control—MetS rat receiving only water; (**C**) heart, MetS 100—MetS rats treated with 100 mg/kg EPP; (**D**) aorta, MetS 100—MetS rats treated with 100 mg/kg EPP; (**E**) heart, MetS 200—MetS rats treated with 200 mg/kg EPP; (**F**) aorta, MetS 200—MetS rats treated with 200 mg/kg EPP. The histological examination revealed no toxic changes in the phenolic-treated rats.

Histology analysis did not reveal any relevant effect of pomegranate peel extract on the anatomical rearrangement in the analyzed tissues. As shown in Table 3, the heart sections were characterized by mild to severe parenchymal degeneration and slightly expressed fibrosis. Inflammatory infiltration was absent in the majority of the samples. The phenolic extract also did not significantly modify aortic fiber disarrangement (Table 4 and Table 5). Representative myocardial and aortic sections are presented in Figure 7.

## 4. Discussion

The current study evaluated the substantial cardioprotective potential of phenolic compounds from pomegranate peels in animal models of MetS, both genetically programmed and induced by diet.

The beneficial health-promoting role of dietary polyphenols in chronic diseases such as MetS has been investigated in many studies. Pomegranate peels seem to be a good source of a wide range of beneficial phytochemicals [36,37]. However, the content of phenolic constituents differs in various pomegranate peel extracts [38]. The quantity of bioactive compounds, in particular, is grounded in the storage conditions, pomegranate cultivar, maturity stage of fruits, and extraction procedure [39]. Polyphenols are susceptible to high temperatures, light, pH, and processing, and all these factors can readily degrade the phenolic content, possibly losing their antioxidant properties [40]. It was proven that the ultimate scope of beneficial phytochemicals is present in pomegranate peels that originated in Turkey [41]. Similarly, *Punica granatum* L. varieties cultivated in Spain are also characterized by a high antioxidative polyphenol content [38]. When comparing the various solvents (i.e., methanol, ethanol, distilled water) used in the extraction procedure, methanol is the most effective in extracting potent antioxidant compounds. However, water and ethanol also seem to be good choices in heterodox studies [42,43].

Based on the studied literature, the ethanolic extract from pomegranate peels (cultivar Mollar de Elche, Spain) used in this investigation possessed valuable phytochemicals [38]. The main class of identified compounds was ellagic acid derivatives, which positively correlate with the high antioxidant properties of the pomegranate peel extract [44]. Precisely, cardiovascular health benefits were accredited to bioactivity exhibited by individual phenolic compounds present in pomegranate peels. Ellagic acid demonstrates an antihypertensive effect, protection against arrhythmia, and cardiac muscle hypertrophy during myocardial infarction [45,46]. It also mitigates doxorubicin-induced cardiotoxicity and ameliorates myocardial diastolic dysfunction occurring in diabetes mellitus [47,48]. Moreover, ellagic acid is accredited to antiatherosclerotic activity by lowering lipid peroxidation [45,49]. The cardiovascular protection of punicalagin evinces itself in moderation of myocardial infarct reperfusion tissue injury, endothelial dysfunction, and pulmonary hypertension [50,51]. Ellagitannins presented at a high concentration in the obtained peel extract as well, contributing to suppressing inflammation, which also plays a crucial role in MetS pathogenesis and cardiovascular consequences [52,53].

Oxidative stress is a crucial component in the pathogenesis of metabolic syndrome and its cardiovascular consequences, including stroke, myocardial infarction, or atherosclerotic disease [54,55]. In obese individuals suffering from MetS, the persistent overproduction of reactive oxygen species (ROS) corresponds with chronic low-grade inflammation affecting miscellaneous organs and results in deleterious effects on their function. Importantly, oxidative stress leads to the formation of insulin resistance in adipocytes, which also plays a vital role in MetS pathophysiology [56]. The imbalance in redox equilibrium aggravates the endogenous enzymatic and non-enzymatic antioxidant defense. The ultimate components of the ROS scavenging system comprise catalase (CAT), superoxide dismutases (SOD), glutathione peroxidases (GPxs), and glutathione transferase (GST) [57]. These antioxidant enzymes provide an oxidation–reduction balance, and the elevation level of these enzymes indicates a boost in the endogenous active oxidative species [58].

In the context of MetS-induced free radical production, the protective influence of the extract from pomegranate peels was observed in the liver and kidneys [59,60]. The antioxidant-promoting effect was demonstrated by reduced oxidative enzyme activity, lipid peroxidation, and increased GSH level in the liver [61,62], as well as decreased reactive oxygen and nitrogen species (ROS/RNS) levels, oxidized LDL (oxLDL) concentration, and SOD activity in the kidneys [59].

The results provided by this study support the hypothesis that phenolic compounds originating from pomegranate peels have superoxide-scavenging activity in myocardial tissue. Especially in terms of the protein thiol group concentration (SH), the molecules marked by the capacity to neutralize the reactive oxygen species were increased in MetS rats treated with pomegranate peel extract in a dose-dependent manner. Data also suggest that ethanolic peel extract increased glutathione disulfide redox buffer in doxorubicin-induced cardiotoxicity [63]. The antioxidative potential of the pomegranate peel extract also enforced an upward tendency inCAT, GPX, and GST activity observed in the rats treated with lower doses, which arise from their mechanism of action. In the presence of reactive oxygen species, these enzymes act as free radical scavengers and convert into inactive forms leading to a decrease in their levels before renewal. Similar results were observed in Wistar rats administered pomegranate peel extract derived from Bosnia and Hercegovina (100 mg/kg BW) for 7 days [64]. As reported previously, the methanol pomegranate peel extract in chlorpyrifos-exposed rats reduces cardiac MDA and increases SOD levels [65]. Our study did not record a favorable influence of ethanol extract from pomegranate peel on their concentration. Total oxidative status (TOS) is useful in assessing the overall redox status [66]. Our findings indicated that a 100 mg/kg BW dose inversely correlated with the TOS concentration. In contrast, in rats treated with 200 mg/kg BW, the alike effect was not determined.

Studies have shown a relationship between pomegranate peel extract supplementation and protection against myocardial tissue failure [25]. As evidenced in isoproterenol-induced myocardial infarction (MI) in Wistar rats, the *Punica granatum* L. peel extract attenuated electrocardiographic changes, myocardial hypertrophy, and lipid peroxidation, and decreased the serum markers of MI. The maximum result was provided with the highest analyzed dosage, 200 mg/kg BW. The upregulation of myocardial expression of endothelial nitric oxide synthase (eNOS), activating nitric-oxide-mediated Nrf2, was concluded to be responsible for the cardioprotection of extract treatment [67]. Similarly, *Punica granatum* L. peel tended to protect the myocardium against toxic agents, demonstrated by the mitigation of ECG disturbances and biochemical parameters [63,65]. Contrary to these outcomes, we did not observe a marked alternation of cTnI level in the rats treated with ethanolic extract. Interestingly, a prominent troponin elevation was detected in healthy rats receiving the phenolic extract. Therefore, it may be concluded that an 8-week EPP exposure might not be enough to moderate the chronic progression of heart failure.

The metabolic consequences also comprise leptin-induced ROS production in heart muscle, leading to lipotoxicity [68]. The results from various studies indicated that *Punica granatum* L. peel extract reduced vascular remodeling and heart tissue damage [67,69]. Our observations did not indicate a marked amendment of the GAL-3 concentration in ethanolic-peel-treated groups, both with MetS and diet-driven obesity. At this point, fibrosis in the histopathological heart section was observed. As mentioned above, the 8-week course of the study might be insufficient for driving collagen fiber deposition in heart tissue. Therefore, the estimation of *Punica granatum* L. peel extract in prevention against myocardial fibrosis linked with cardiac failure requires further investigations.

The implications of obesity and MetS in cardiovascular tissue reconstruction incorporate a wide range of pathologies, including cardiomyocytes necrosis, interstitial fibrosis, increase in connective tissue deposition, myocardial hypertrophy, adipose tissue infiltration, and coronary vessels sclerosis and narrowing [70,71]. Alternations in cardiac muscle equal its stiffness and diastolic dysfunction [72]. The proposed molecular mechanism of heart failure is pyroptosis, marked by inflammatory-regulated cell death triggered by metabolic alternations [73]. Evidence has shown that pomegranate peel extract can reduce the hypertrophied coronary arterial walls induced via the downregulation of angiotensin-converting enzyme activity (ACE) [69]. Of note, referring to cardiac muscle, phenolic extract from peels improved myofibril composition via the reduction in separated and disorientated muscle fibers with pyknotic nuclei, inhibition in cytoplasmatic lipid deposition, and vacuolar degeneration of cardiomyocytes [63,67,74]. In our study, histopathological data revealed considerable alternation, such as parenchymal degeneration in heart sections and fiber displacement in the aorta cross-sectional area in all the groups of rats, which are linked to the course of metabolic imbalance, especially co-occurring obesity and diabetes mellitus in MetS. The administration of EPP did not result in notable improvements and reduction in structural variations. However, in an animal model of streptozotocin-induced diabetic cardiomyopathy, the administration of pomegranate peel extract (150 mg/kg BW) exhibited a significant reduction in myocardial fibrosis, reflected by lowering the cardiac myofibrils reactivity against TGF-β and collagen fiber deposition [75]. No necrosis or toxicity effect in cardiomyocytes in all EPP-administered groups showed that ethanol extract from pomegranate peels did not lead to myocardium damage, and its usage is safe in the context of the cardiovascular system.

Albeit, some limitations of the study must be taken into account. Primarily, each group of the MetS rats consists only of six individuals. Nonetheless, before our research, the minimal sample size in terms of the analyzed values was calculated to warrant statistically relevant data. Reduction in the scrutinized population refers to the principle of the 3Rs in animal experimentation (replacement, reduction, and refinement), improving the welfare of animals used in science [76]. The EPP composition is not standardized, and the phenolic constitution, both quantitative and qualitative, depends on miscellaneous variables, such as the fruit cultivar used, maturity stage, and storage conditions, which also act as a disadvantage. Finally, even though Zucker diabetic rats proved to be a suitable animal model for metabolic syndrome, further double-blinded and randomized trials are essential for proving Punica granatum L. peel bioavailability and efficacy in humans.

## 5. Conclusions

The observations presented in this study support the hypothesis that pomegranate peels play a role as an antioxidant agent in heart tissue. The polyphenolic extract had a significant impact on the protein thiol group concentration (SH) and total oxidative status (TOS). The influence on inhibiting the myocardium architecture remodeling and the myofibril necrosis reflected by troponin I (cTnI) and galectin 3 concentration (GAL-3) was not confirmed. Importantly, no toxic effect of the applied dosages on cellular integrity and tissue structure in the heart and aorta sections was observed. These findings support the beneficial role of pomegranate peel phenolic composition as a nonhazardous, antioxidative agent in heart tissue. However, to comprehensively divulge the usability of phenolic constituents extracted from pomegranate peels, further studies on humans must be conducted to determine the real nutritional and medical value.

## Figures and Tables

**Table 1:** Mass spectrum characteristic and content of phenolic compounds in pomegranate peel extract.

T pRt	MS [M-H]^-^ (*m*/*z*)	MS/MS [M-H]^-^ (*m*/*z*)	Name of Compound	Polyphenol Content
1.67	331	271/169	Galloyl-glucose	2.00 ± 0.03
1.73	781	721/601	Punicalin α/A	3.11 ± 0.06
2.02	1083	611/331/146	HHDP-galloyl-hexoside (punicalagin)	4.20 ± 0.09
2.12	1083	781/622/301	Punicalagin isomer	14.82 ± 1.04
2.33	933	631/450/301	Ellagitannin	4.71 ± 0.40
2.87	1083	781/301	HHDP-gallagyl-hexoside (punicalagin)	93.91 ± 2.05
3.12	1085	907/783/301	Ellagic acid derivative	2.49 ± 0.53
3.69	1083	781/301	HHDP-gallagyl-hexoside (punicalagin)	157.0 ± 2.65
3.89	799	301	Granatin A	4.74 ± 0.32
5.08	783	481/301	Ellagitannin	25.86 ± 1.53
6.20	1085	933/301	Digalloyl-gallagyl-hexoside	10.37 ± 0.65
6.25	783	481/301	Ellagitannin	13.51 ± 0.99
6.38	463	301	Ellagic acid-hexoside	33.63 ± 1.23
6.89	951	907/635/301	Galloyl-HHDP-DHHDP-hex (granatin B)	2.68 ± 0.11
			Total (mg/g dw)	373.05

**Table 2:** Determination of oxidative stress marker concentration in the heart tissue by the group. Groups: MetS control (only water), MetS 100 (EPP at a dose of 100 mg/kg BW), and MetS 200 (EPP at a dose of 200 mg/kg BW). The data are displayed as the mean ± standard deviation (SD).

	MetS Control	MetS 100	MetS 200	p_ANOVA_	p_control_ _vs. 100_	p_control_ _vs. 200_
CAT (IU/g protein)	46.1 ± 3.14	56.7 ± 2.1	48.2 ± 3.0	<0.001	<0.001	0.364
GR (IU/g protein)	11.0 ± 1.4	12.0 ± 1.2	10.2 ± 1.2	0.063	–	–
TOS (μmol/g protein)	0.31 ± 0.09	0.09 ± 0.01	0.26 ± 0.05	<0.001	<0.001	0.227
SH (μmol/g protein)	2.2 ± 0.4	4.1 ± 0.6	8.5 ± 0.9	<0.001	<0.001	<0.001
SOD (NU/mg protein)	40.0 ± 1.4	41.6 ± 1.6	40.9 ± 3.1	0.455	–	–
MnSOD (NU/mg protein)	37.6 ± 1.1	39.2 ± 1.5	39,7 ± 2.5	0.155	–	–
GPx (IU/g protein)	6.1 ± 0.9	7.0 ± 0.4	5.9 ± 0.9	<0.05	0.071	0.876
GST (IU/g protein)	0.99 ± 0.13	1.27 ± 0.18	1.00 ± 0.16	<0.05	<0.05	0.991
MDA (μmol/g protein)	6.5 ± 0.7	6.1 ± 1.1	6.3 ± 1.4	0.790	–	–

Abbreviations: CAT—catalase, GR—glutathione reductase, TOS—total oxidative status, SH—protein thiol groups, SOD—superoxide dismutase, MnSOD—Mn-dependent superoxide dismutase, GPx—glutathione peroxidase, GST—glutathione transferase, and MDA—malondialdehyde.

**Table 3:** Determination of heart failure markers concentration in heart tissue (GAL-3) and in serum (cTnI) by the group. Groups: MetS control (only water), MetS 100 (EPP at a dose of 100 mg/kg BW), and MetS 200 (EPP at a dose of 200 mg/kg BW). The data are displayed as mean ± standard deviation (SD).

	MetS Control	MetS 100	MetS 200	p_ANOVA_
cTnI (pg/mL)	338.3 ± 92,1	359.1 ± 125.3	501.2 ± 97.9	0.244
GAL-3 (pg/mL)	30.7 ± 1.1	30.3 ± 0.9	30.7 ± 0.9	0.670

Abbreviations: cTnI—troponin I, GAL-3—galectin 3.

**Table 4:** Evaluation of changes in the heart specimens on a semi-quantitative scale (0—no changes, 3—severe changes. Groups: MetS control (only water), MetS 100 (EPP at a dose of 100 mg/kg BW), and MetS 200 (EPP at a dose of 200 mg/kg BW).

	MetS Control	MetS 100	MetS 200
Parenchymal degeneration	1 (0–2)	2 (2–3)	2 (1–3)
Fibrosis	0 (0–0)	0 (0–1)	0 (0–2)
Inflammatory infiltration	0 (0–0)	0 (0–0)	0 (0–3)

**Table 5:** Evaluation of changes in aorta specimens in a semi-quantitative scale (0—no changes, 3—severe changes). Groups: MetS control (only water), MetS 100 (EPP at a dose of 100 mg/kg BW), and MetS 200 (EPP at a dose of 200 mg/kg BW).

	MetS Control	MetS 100	MetS 200
Fiber disarrangement	0 (0–1)	1 (1–3)	1 (0–2)

## Data Availability

The data that support the findings of this study are available on request from the corresponding author. The data are not publicly available due to privacy and ethical restrictions.
